# Auditory traits of "own voice"

**DOI:** 10.1371/journal.pone.0199443

**Published:** 2018-06-26

**Authors:** Marino Kimura, Yuko Yotsumoto

**Affiliations:** Department of Life Sciences, The University of Tokyo, Tokyo, Japan; Tokai University, JAPAN

## Abstract

People perceive their recorded voice differently from their actively spoken voice. The uncanny valley theory proposes that as an object approaches humanlike characteristics, there is an increase in the sense of familiarity; however, eventually a point is reached where the object becomes strangely similar and makes us feel uneasy. The feeling of discomfort experienced when people hear their recorded voice may correspond to the floor of the proposed uncanny valley. To overcome the feeling of eeriness of own-voice recordings, previous studies have suggested equalization of the recorded voice with various types of filters, such as step, bandpass, and low-pass, yet the effectiveness of these filters has not been evaluated. To address this, the aim of experiment 1 was to identify what type of voice recording was the most representative of one’s own voice. The voice recordings were presented in five different conditions: unadjusted recorded voice, step filtered voice, bandpass filtered voice, low-pass filtered voice, and a voice for which the participants freely adjusted the parameters. We found large individual differences in the most representative own-voice filter. In order to consider roles of sense of agency, experiment 2 investigated if lip-synching would influence the rating of own voice. The result suggested lip-synching did not affect own voice ratings. In experiment 3, based on the assumption that the voices used in previous experiments corresponded to continuous representations of non-own voice to own voice, the existence of an uncanny valley was examined. Familiarity, eeriness, and the sense of own voice were rated. The result did not support the existence of an uncanny valley. Taken together, the experiments led us to the following conclusions: there is no general filter that can represent own voice for everyone, sense of agency has no effect on own voice rating, and the uncanny valley does not exist for own voice, specifically.

## Introduction

“Who am I?” This question, which is at the heart of the sense of self, has been asked and challenged for a long time by artists, philosophers, and scientists [1–4]. To measure the conceptual “self” scientifically, the sense of “self” has been represented using several modalities as stimuli. The self-face is the most frequently used experimental stimuli due to its representativeness and convenience. Although most self-focused psychological experiments have used self-face, one’s voice is also an important component of “self.” Indeed, one does not witness one’s own face except on horizontally flipped images on mirrors. However, humans are frequently exposed to their own voice suggesting it may be a better, more representative example of real world self-representation.

Speech sounds are produced in the vocal fold and delivered to the vocal cavity. They then travel to the ear and auditory nerve via an air-conducted pathway from the mouth and a bone-conducted pathway via the cranial bones [5]. The bone conduction pathway also includes soft tissues. These different forms of sound conduction result in the different sounds and manifestations of hearing. Even though one can recognize if the presented voice is theirs, the recorded voice is found to be very unlike the voice that one hears when they are speaking. This is because the voice that one hears (own-voice) includes both bone conduction and air conduction while the recorded voice only includes air conduction [6,7]. In addition, air conduction may also be distorted in the recorded voice, because the recorded voice is recorded close to the mouth, while own voice is “played” in the mouth. Further, depending on the audio set up, recorded voice may originate closer or farther from the ear than spoken voice. This difference may also contribute to the difference between own voice and recorded voice.

Over decades, researchers of the transfer function in own voice have employed various experimental methods. For example, the resonance frequencies of the human skull of patients with skin penetrating titanium implants were measured [8]. Bone transfer functions have been estimated using distortion product otoacoustic emissions [9]. Finally, the frequency characteristics of four different bone conduction actuators have been investigated [10]. Based on bone conduction characteristics described in previous research, the equalization filter is considered a suitable method to reproduce own-voice from recorded voice. Although filtered voice was rated as own-voice rather than recorded voice, the filter types varied across studies [11–13]. Moreover, differences in the experimental settings, e.g. the words used as stimuli, impede the direct comparison of experimental results.

As previous studies were only concerned with frequency cut-off filters, the possible contributions of other sound characteristics, such as vibrato and pitch, as a component of own voice have not yet been examined. As some people tremble when they speak, instability of the voice may affect own-voice perception. Voice instability corresponds to vibrato, as they share characteristics [14]. Pitch may be another specific trait of own voice. Poor-pitch (i.e. tone deaf) singers have difficulty in mapping pitch onto action, but perceptual, motor, or memory problems have not been found in these individuals [15]. When the speaker tries to reproduce required pitch sounds, the speaker may have recognized bone conducted own voice as the correct pitch resulting in “poor-pitch”.

Other than sound characteristics, sense of agency is said to be an important component of self-ness. The online sense of action performance (“I am the one who is causing action”) is referred to as sense of agency, in which the performance done by someone else is being distinguished [16]. Sense of agency does not only concern body movement but also speech monitoring of auditory perception. It is known that mouth movement during sound presentation induces a higher sense of agency than images or hearing alone [17]. The effect of sense of agency presence on own voice, whether it encourages or changes the own voice representation within one’s self, has not been investigated.

There are strong links between speech acoustics and emotions [18,19]. Listeners are able to perceive the intended emotions from spoken voices, indicating that listeners associate particular patterns of acoustic cues with various discrete emotional states, and that the ability to infer emotion from speech is a fundamental component of human vocal communication [20]. Besides the profound relationship between emotion and voice [21], perception of voices is also critical in various situations. For example, newborn infants clearly prefer their mother’s voice [22,23], and voice-only communication elicits greater empathy [24]. Furthermore, recent technology developments have increased the demand to use human-like voice in vocal assistance robots. A number of studies have examined how synthesized robotic voices are perceived by humans [25,26], and explored the best form of user-friendly acoustic interfaces. Despite the importance of voice perception in the human interactions, as well as human-machine interfaces, we are yet to fully understand how we perceive our own voices. Hence, it is critical to precisely evaluate the perception and representation of own-voice.

**Fig 1:**
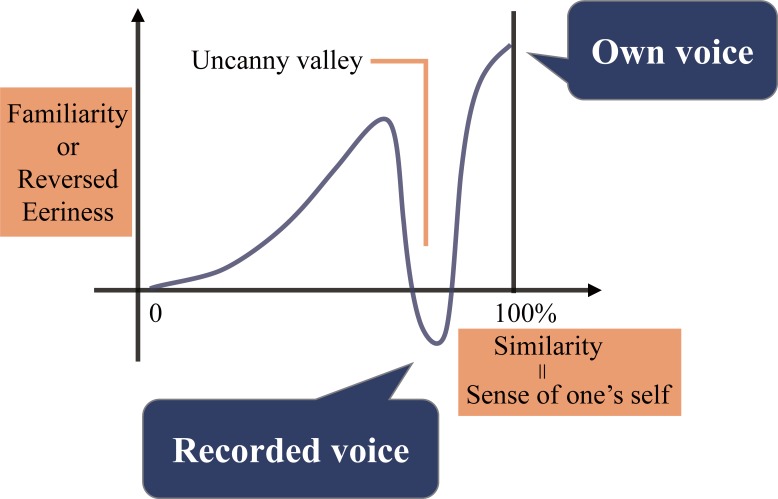
Conceptual diagram of the uncanny valley in the voice field. **Adapted from “The Uncanny Valley,” by M. Mori, 1970.** Conceptual diagram of the theoretical graph presented in the original uncanny valley theory. X-axis corresponds to similarity between robots and humans and y-axis corresponds to familiarity of the robots. Recorded voice may represent the valley part and own voice the highest point after the valley. Sense of one’s self instead of similarity was used in the present study.

In addition to own voice reproduction, we also focused on differences in discomfort between own-voice and recorded voice. Even though most people may judge the presented voice as own-voice, non-modified recorded voice is found to be unpleasant. This phenomenon may be due to the recorded voice creating a so-called the uncanny valley (Fig 1). The uncanny valley is a widely used concept first proposed in the field of robotics [27]. The idea claims the familiarity and empathy to humanlike robots increases as the appearance of the robot becomes similar to human beings. However, in robots very closely approximating but failing to attain human appearance, the response by humans turns into revulsion. As an explanation, the original theory stated, “eeriness can be represented by negative familiarity.” Previous studies investigating the existence of the uncanny valleys have used eeriness, familiarity, and humanlike-ness as measurements [28,29].

Our first experiment investigated the consistency of own-voice rating and queried which equalization filter among those employed in previous studies best represents one’s own voice in a controlled experimental setting. The filters compared were: one that attenuated and amplified a certain range of frequency, one that cut off frequency at a strict threshold, and one that omitted a certain range of frequency. In addition to the filter comparison, the possibility of contributions from other sound characteristics, such as pitch and vibrato, to one’s own voice representation was examined. In experiment 2, we examined the effect of sense of agency on own-voice representation by activating the motor system. Finally, in experiment 3, we measured familiarity, eeriness, and sense of one’s self to investigate the existence of the uncanny valley in the acoustic field, focusing on each individual's voice features.

## Experiment 1

### Introduction

In experiment 1, the sound profile that best represents own voice was examined. We used filters described in previous studies, as follows: +3 dB for a signal higher than 1 kHz and -3 dB for a signal lower than 1 kHz as a step filter [11]; a trapezoid like filter as a lowpass filter [12]; filter passing from 300 to 1200 Hz as a bandpass filter [13]. In addition to these three types of filters, an adjusted voice protocol, in which the participants adjusted all or part of pitch, vibrato, and frequency cut off filters of recorded voice to reproduce own-voice, was added for comparison. The participants chose the stimulus that best represented own-voice by comparing recorded voice, step filtered voice, lowpass filtered voice, bandpass filtered voice, and adjusted voice. To examine the consistency of the own-voice rating, the participants rated own-voiceness twice on two different days.

### Methods

#### Participants

Ten Japanese students (four females and six males, 18–22 years old) who reported no hearing disorders were paid to participate in the experiment. All participants gave written informed consent in accordance with the Declaration of Helsinki for their participation in the experimental protocol, which was approved by the institutional review board at The University of Tokyo.

#### Apparatus

Each participant’s voice was recorded in a soundproof room using Sennheiser Microphone ME62 (Sennheiser electronic GmbH & Co.KG, Germany) and Focusrite audio interface (Scarlett 2i4, First Generation model; Focusrite, UK). Audacity, downloaded from www.audacityteam.org, was used to save a digital recording of the voice. All recorded voice was digitized at a 16 bit/44.1 kHz sampling rate. The auditory stimuli were presented through a USB digital-to-analog converter Focusrite audio interface Scarlett 2i4 1^st^ Generation and MDR-XB500 headphones at 60 dB (SONY, Japan). The visual stimuli were presented on a LCD monitor (BenQ, China) using MATLAB R2015b (The MathWorks, Inc., USA) and the Psychtoolbox (www.psychtoolbox.org). The open-source patch DAVID (Da Amazing Voice Inflection Device)[21] for the close-source audio processing platform Max (Cycling ‘74, USA) was used to allow participant control of auditory features of voice in real-time.

#### Stimuli and procedure

The experiment consisted of three sessions with the protocol for each filter setting conducted on 3 individual days. In session 1, the voice was recorded and the parameters of the voice were modified. Twenty-six three-syllable Japanese words categorized as neutral were selected [30] and recorded as the stimuli. The participants pronounced the stimuli in their usual manner. The participants were instructed not to correct their dialects. After the recording of all 26 words, the participants freely modified filters for pitch, vibrato, and frequency features of the original voice (recorded voice) such that the recording sounded like the voice that they hear when speaking (own voice). The participants were given the instruction of how to use graphical user interface for modification. The experimenter sat aside of each participant, and instructed the usage of GUI step by step until the participant fully understood the procedure. After this training period, the participants underwent the actual experimental trials. They were allowed to take time as long as they needed until they were convinced that the adjusted voice was their own. Vocalization was neither restricted nor encouraged while the participant modified the parameters of the voice. To control familiarity to the stimulus, six words of the recorded voices were used in this voice adjustment phase, and the remaining 20 recorded voices were used later in the rating phase; i.e., words used in the voice adjustment phase were not used in the rating phase in order to control for familiarity of the rated words.

**Fig 2:**
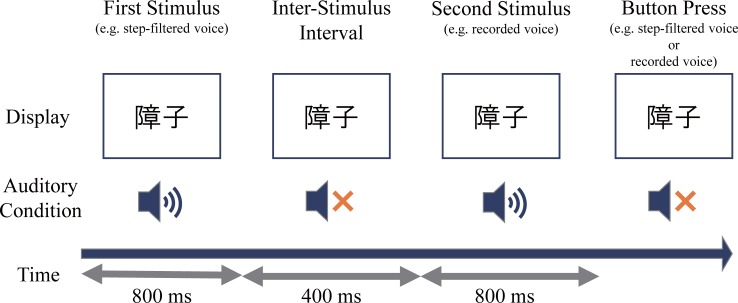
Experiment 1. **Schematic of the task.** After the presentation of stimuli, participants chose which of the stimuli sounded more like own-voice by button press.

In sessions 2 and 3, the participants were asked to participate in the voice rating task, and the exact same procedures were repeated. The participants performed two alternative forced choice tasks that involved listening to two different voice conditions and answering which voice sounded more like their own voice (Fig 2). The voice conditions that were judged included: recorded voice, step filtered voice, bandpass filtered voice, lowpass filtered voice, and adjusted-by-will voice (adjusted voice). In order to control the individual difference of own voice perception and to prevent individual variability in the rating procedure, stimuli were presented as a pair to force participants to decide which of the presented stimuli sounded more like own voice. Each of the five voice conditions was paired with another condition in each trial. Combinations of five filters with counterbalanced presentation orders resulted in 20 pairs of the filters. Each pair of the filters was tested with the 20 words prepared for the rating phase. As a result, 400 trials were conducted in the rating phase. All 400 trials were randomized within the session. Inter-stimulus interval was fixed as 400 ms and each stimulus was 800 ms. Within a trial, each stimulus was presented only once without repetition. There were 10 blocks in one session, each block containing 40 trials. Participants were able to take a break between the blocks.

#### Analysis

The own voice ratings were analyzed by a pairwise comparison method [31], which enables plotting of the scores of each condition on the same scale, so that each participant’s relative preference could be evaluated. Thurstone’s pairwise comparison method ranks the responses based on the z values calculated from the percentage of the choice of each item. For all pairs of recorded, step-filtered, lowpass-filtered, bandpass-filtered, and adjusted voices, the proportion of the stimuli chosen as a more own-voice like sound was calculated. The inverse function of the standard normal distribution was calculated and averaged for each stimulus. Then, each participant’s own-voice rating was schematized into a scale bar. To evaluate the consistency of own-voice rating for a participant, Spearman’s rank correlation coefficient was also calculated across two sessions carried out on two independent days.

### Results and discussions

We verified that voice transformation with DAVID worked as the participants intended, by analyzing the pitch of modified and non-modified speech samples using the SWIPE algorithm [32], and confirmed that actual pitch differences matched the parameter settings saved by the participants (see S1 Fig, S2 and S3 Tables).

**Fig 3:**
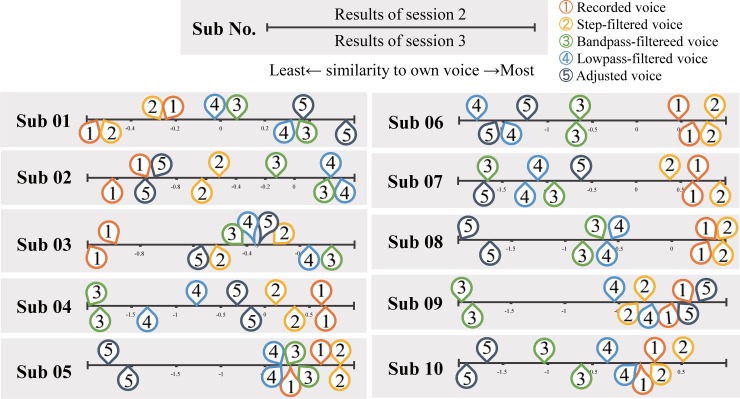
Experiment 1. **Individual results of pairwise comparison.** The bar represents the similarity to own voice, rightmost represents the most own-voice like and leftmost represents the least own-voice like rating. The numbers on the top-half of the bar represents the result of the second session and the ones on bottom-half of the bar are the results of the third session. The numbers are for types of conditions: 1) Recorded voice, 2) Step filtered voice, 3) Bandpass filtered voice, 4) Lowpass filtered voice, 5) Adjusted voice.

Individual results of pairwise comparisons are shown in Fig 3 and S1 Table. The voice rated as most similar to own voice differed across participants. Two participants chose the recorded voice most representative of own voice, and eight participants rated modified voice as most like own voice. Individual differences were found in the own voice rating, indicating there was no general filter that represented own voice. Even though each participant adjusted part or all of the pitch, vibrato, and frequency cut off filter to sound like own-voice (see S2 Table for details), only Sub 01 and 09 rated the adjusted voice as the own-voice. The various availabilities of modifiable parameter choices may have confused participants, resulting in prolonged adjustment times that made participants tired. There is also a possibility of participants unknowingly vocalizing the own voice closer to the recorded voice as part of their review of own voice.

**Fig 4:**
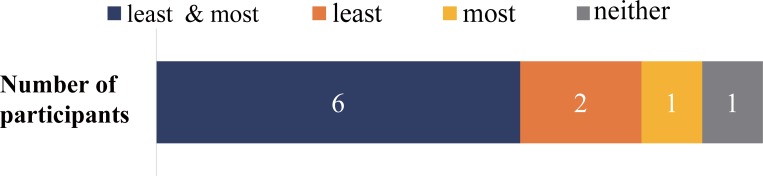
Experiment 1. **Consistency of own-voice rating across trials.** The consistency of the most and the least own-voice like rating is presented. Blue represents the number of participants who rated both the most and least own voice-like voice consistently, orange represents the number of participants who rated only the least own voice-like sound consistently, yellow represents the number of participants who rated only the most voice-like sound consistently, and gray represents the number of participants who rated both the most and the least own voice-like sound inconsistently.

Fig 4 represents the consistency of similarity to own-voice rating across days. Six participants rated the voices the least and the most similar to own voice consistently, two participants rated the least own voice representative condition consistently, and one participant rated the most own voice representative condition consistently, while one participant showed no congruence. Spearman’s rank correlation coefficient calculation across the participants revealed high rank correlation of most (*ρ* = .899) and least (*ρ* = .900) own-voice ratings between the two different sessions done on two different days. The result suggests that the perception of own-voice was steady to a certain extent across experimental days.

## Experiment 2

### Introduction

Whenever one listens to their own voice, the mouth is moving due to speaking. In experiment 1, the participants did not move their mouth during voice rating, creating a difference from the actual vocalizing environment. Lack of mouth movement may have resulted in decreased sense of agency. The aim of experiment 2 was to investigate if mouth movements during hearing one’s voice affects own-voice rating. Thus, an additional day was added to the experiment 1 paradigm in which participants were asked to move their mouth at the same time as they heard their voice.

### Methods

#### Participants

Seven subjects from experiment 1 returned to participate in experiment 2, and nine additional participants were recruited. In total, 16 Japanese students (11 males and five females, 18–22 years old) who reported no hearing disorders were paid to participate in the experiment. Payments were transferred to the subjects’ bank accounts after participation. All participants gave written informed consent in accordance with the Declaration of Helsinki for their participation in the experimental protocol, which was approved by the institutional review board at The University of Tokyo. The data of one participant was excluded due to data corruption caused by Wi-Fi disconnection during the experiment.

#### Apparatus

The experimental apparatus was same as that in experiment 1.

#### Stimuli and procedure

Experiment 2 consisted of four sessions, each of which was conducted on 4 different days. Session 1 was assigned for voice recording and parameters were adjusted as in experiment 1. All or part of pitch, vibrato, and frequency cut off filters were adjusted. Three remaining sessions were assigned to rate which of two presented stimuli sounded more like own voice by two alternative forced choice tasks. The five conditions (recorded voice, step-filtered voice, bandpass-filtered voice, lowpass-filtered voice, and adjusted voice) were presented and judged in the task. Each of the five voice conditions was paired with another condition in each trial. Permutations of five filters were taken two at a time resulting in 20 total trials. Twenty words were rated in this experiment, resulting in 400 trials in total. All the 400 trials were randomized within the session. Inter-stimulus interval was fixed as 400 ms and each stimulus was 800 ms. Within a trial, each stimulus was presented only once without repetition. There were 10 blocks in one session, with each block containing 40 trials. Participants were able to take a break between blocks.

One of the three sessions was assigned as a lip synchronization session while the other two sessions were non-lip synchronization sessions. In the lip synchronization session, the participants were asked to synchronize their lips without vocalization as the stimulus was presented with the corresponding letter on the screen. On each trial, two stimuli were presented as a pair and the participants synchronized their lips for both stimuli. A training session with twenty trials was conducted prior to the actual experimental trials. In the training phase, the voices used in the parameter adjustment phase were used, so that practice did not affect familiarity of the rated word. The order of the lip synchronization session and non-lip synchronization sessions were counter-balanced across participants.

#### Analysis

Experimental analysis was same as in experiment 1.

### Results and discussions

**Fig 5:**
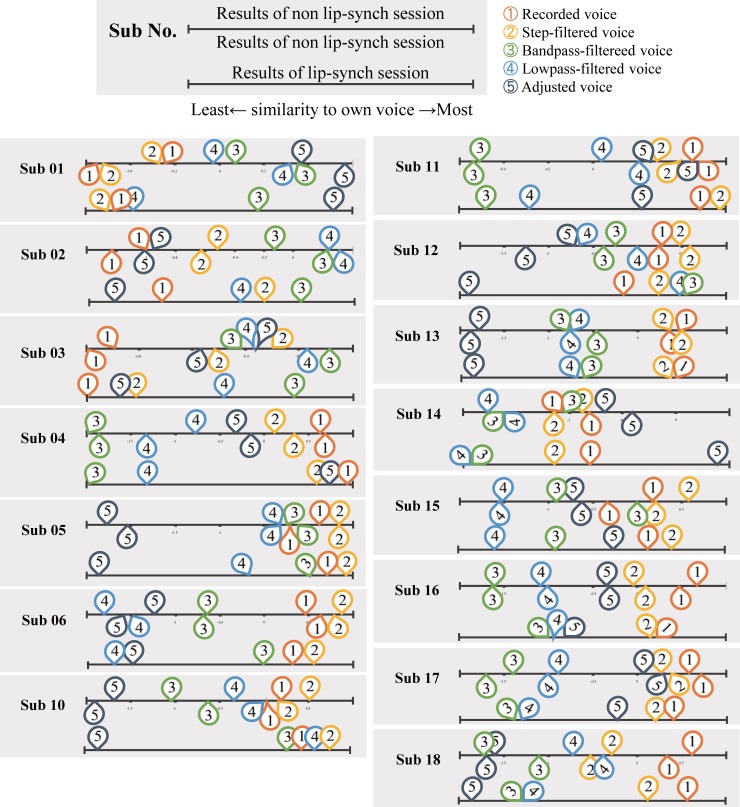
Experiment 2. **Individual results of pairwise comparison.** The bar represents the similarity to own voice, rightmost as the most own-voice like and leftmost as the least own-voice like rating. There were two non-lip synchronization sessions conducted and the results are presented as the numbers on the top bar. The numbers on the bottom bar represent the results of the lip synchronization session. The numbers are the types of conditions: 1) Recorded voice, 2) Step-filtered voice, 3) Bandpass filtered voice, 4) Lowpass filtered voice, 5) Adjusted voice.

The results of pairwise comparison on individual data are shown in Fig 5 and S1 Table. Similar to that of experiment 1, insomuch as individual ratings largely differed. Each of the voice manipulations was chosen as the most own-voice like by at least one of the participants, including the non-manipulated voice which was preferred by 4 of the participants. Four others rated the recorded voice the highest for own-voice similarity. The results of the lip synchronization session resemble those of the non-lip synchronization sessions, few participants chose the adjusted voice as the most own voice representative. It should be noted that adjustments of various parameters that participants were unfamiliar to might have caused confusion and tiredness.

**Fig 6:**
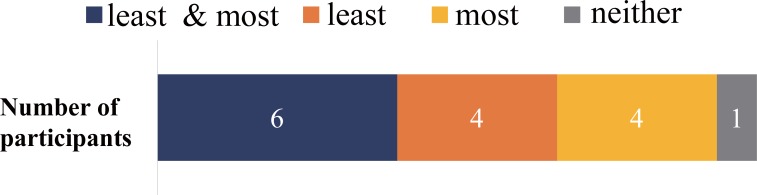
Experiment 2. **Participant own voice rating consistency across days.** The consistency of own voice-like rating across participants is charted. Blue represents the number of participants who rated both the most and least own voice-like voice consistently, orange represents least choice consistency only, yellow represent most choice consistency only, and gray represents inconsistency for both the most and least own voice-like voice.

The consistency of own voice similarity rating across sessions is shown in Fig 6. Six participants rated both the least and most own-voice representative consistently, eight participants rated either the least or the most consistently, and one participant rated inconsistently. High rank correlations of the most (*ρ* = .899) and the least (*ρ* = .900) own voice across three different sessions were shown by Spearman’s rank correlation coefficient calculation across the participants. The results showed the voice chosen for best own-voice representative was consistent within participant but not across participants, meaning own voice depiction remains consistent within individuals.

Thus, lip synchronization, which aims to increase sense of agency by replicating motor control during vocalization, may not affect own voice rating. There were only two participants who changed the rating of the least own-voice-like condition on the lip synchronization session and two who changed the most own-voice-like rating. Lip synchronization may not affect own-voice perception, at least in our experimental paradigm.

## Experiment 3

### Introduction

Experiments 1 and 2 showed the consistency of own-voice rating for individuals and the difference among individuals for own-voice perception. In experiment 3, we examined how own-voice relates to the uncanny valley. The participants were asked to rate how much the voice sounded like own-voice (sense of one’s self), familiarity, and eeriness of each stimuli.

### Methods

#### Participants

Twelve Japanese students (seven males and five females, 18–24 years old) who reported no hearing disorders were paid to participate in the experiment. All participants gave written informed consent in accordance with the Declaration of Helsinki for their participation in the experimental protocol, which was approved by the institutional review board at The University of Tokyo.

#### Apparatus

The experimental apparatus was same as the one in experiments 1 and 2.

#### Stimuli and procedure

Experiment 3 consisted of two sessions and was conducted on 2 different days. In session 1, the voice of each participant was recorded, and participants adjusted parameters to make the recorded voice sound like their own voice as in Experiments 1 and 2. The words used as stimuli in experiment 3 were the same as those in experiment 1 and 2. The voice conditions were also the same; recorded, step-filtered, lowpass-filtered, bandpass-filtered, and adjusted voice.

**Fig 7:**
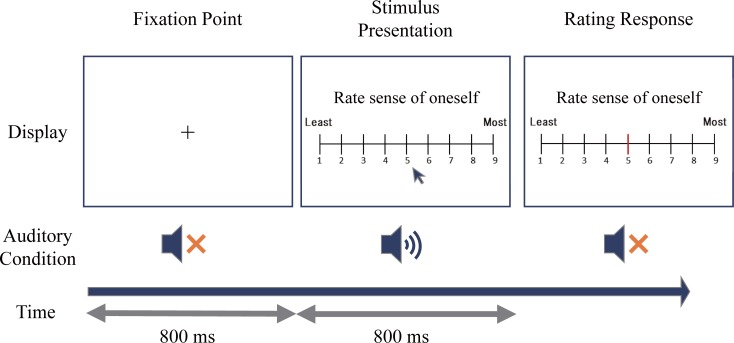
Experiment 3. **Schematic of the experimental task.** After the presentation of stimulus, the participant rated the stimulus in terms of the presented feature from one to nine by moving a cursor.

The rating of the presented voice was conducted in session 2. The session consisted of three blocks, each block containing a different type of rating; familiarity, eeriness, and sense of oneself. The order of the blocks was counter-balanced across participants. In each trial, an 800 ms fixation cross was presented followed by 800 ms of stimulus presented through the headphones. The task was to rate either the sense of oneself, familiarity, or eeriness of the presented voice. The participants made responses by moving a cursor over a 9-poinit Likert scale (Fig 7). The black cursor, corresponding to chosen score, turned red after a click. In each trial, the stimulus was played only once and there was no option of replay. The participants were instructed to rate familiarity, eeriness, or sense of oneself, using the whole scale to be as precise as possible. After 30 practice trials, the participants completed all 300 trials. The participants took a break between each block.

#### Analysis

After standardization of the corrected data, a cubic equation was used to examine if sense of oneself relates to the uncanny valley. Our research is the first study focusing on the voice representation in the uncanny valley theory, as humanoid face context had been the most researched topic, the analysis herein was exploratory. We therefore investigated if the presented stimuli covered the whole range from 0 to 100% similarity or part of the range, such as from 80 to 100%. In addition, the mode of the posterior distribution of the correlation coefficient was calculated with Bayesian statistics to assess if sense of oneself or familiarity showed a correlative relationship with the eeriness of the voice. In addition to the coefficient between two scores, average and variance of each score were treated as unknown parameters to estimate posterior distribution. The distribution of correlation was sampled by Markov-chain-Monte-Carlo calculation, and the mode of the posterior distribution was treated as the correlation coefficient.

### Results and discussions

**Fig 8:**
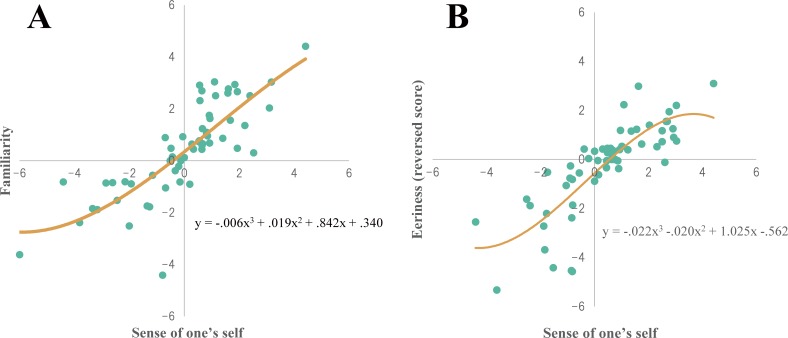
Experiment 3. **Results of voice features scoring.** The X-axis represents sense of oneself, y-axis represents familiarity for A and eeriness for B. Each individual score is plotted as green dots. The dotted line shows the Pearson’s correlation and the solid represents the cubic equation.

Fig 8 shows scores of familiarity, eeriness, and sense of oneself of all five conditions in scatter plots. The eeriness score was plotted in reverse, higher scores indicating lower eeriness, as shown in Fig 8B. Bayesian statistics showed a strong positive correlation for both familiarity and sense of oneself (*r* = .807), and eeriness and sense of oneself (*r* = .803). In addition, the cubic equation calculated from the data drew a gradual undulating curve for both familiarity and sense of oneself, and eeriness and sense of oneself. While there were strong positive correlations, the slope exhibited a constant increase without indicating the existence of an uncanny valley. Thus, the expected extreme valley proposed by Mori (1970) was not observed.

## General discussions

In the present study, we explored the best modification protocol for reproducing own voice from recorded voice, and investigated the effect of the motor system on own voice representation. The voice manipulation perceived as most similar to the own voice varied substantially between individuals, but was consistent across separate days. In addition, we found no effects of lip-synching on own voice perception. The correspondence of recorded voice familiarity to the uncanny valley was also examined in this study. Although proportional relationships were found between familiarity and sense of oneself, and between eeriness and sense of oneself, evidence to support the existence of the uncanny valley was insufficient.

Beside the attempts to find one generalized filter to reproduce own voice, our study indicated that there was no such universal filter. The sound chosen as the best own voice representative differed greatly across the participants. No specific filter or any specific modification of acoustic traits could be applied to everyone to make recorded voice sound like own-voice. A previous work suggested a band-pass filter to be universal [13]. There are two major differences between their study and the present study. First, the previous work examined voice perception of singers, who are more likely to be exposed to their own-voices, while the participants of the present study had no particular training prior to this experiment. Second, the previous study used 'Ah' in the sung voice as the parameter for manipulation, whereas we used six words selected from the vocabulary used in our daily life. Therefore, we believe our results are more generalizable. Differences in body structures or experience of exposure to recorded voice may be considered as reasons for individual differences. As no human being is identical to someone else, everyone’s voice is distinct. Some people, such as actors or singers, listen to their recorded voices in daily life, but some rarely listen to their recorded voices as much as their own voices. This difference of own-voice exposure frequency may result in individual differences in own voice perception.

Along with the individual differences found in own voice perception, the stability of own voice perception within individuals was explored. Although people now observe their faces not only in the mirror but via easily taken photographs on mobile devices, own voice is still the most frequently perceived self-representing feature. As people listen to their own voice countless times in their daily lives, the perceptions of own voice may become solid and robust.

In the context of own voice reproduction, bone conduction was thought to be the most important component in addition to air conduction. A recent study proposed that the bone is not the only substrate in own voice conduction, as cartilage is now discussed as a third sound transmission pathway [33]. The aural cartilage is part of the outer ear and covers half of the exterior auditory canal. The differences between transmission mechanisms result in differences in hearing, cartilage conduction produces a broader sound range and stereophony [34]. It should be noted that our experimental procedure evaluated the sound transmission pathway including bone conduction as well as cartilage conduction. Therefore, we were unable to dissociate the effect of bone conduction and cartilage conduction from our result.

It is said that the human body conducts low and rich tones, and people often claim they perceive their own voice to be lower and richer in tone than their recorded voice. Despite these phenomena, some people have reported the recorded voice to be higher than modified voices. Our study allowed detailed and independent modulation accounting for such individual differences, but the voice adjusted by each participant was not necessarily the most representative of own-voice. This might be because the modification of voice characteristics induced changes in emotional impressions of the voice, as previous studies showed that slight modifications of voice parameters cause changes of emotion rating in a congruent direction [35]. There is a possibility of some emotional characteristics of adjusting the voice recording distracting from own-voice perception, such that absolute rating of adjusted voice differs from relative rating.

Our study used lip-synching during voice presentation in order to consider the effect of sense of agency on own-voice rating. There was no effect of lip-synching on sense of agency in own voice perception. This may owe to issues with the experimental procedure. Instructions to move the mouth at the same time as voice presentation with completely random and various vocabularies may have produced time lag between auditory perception and sense of agency. Moreover, the possibility of unpredictability and mismatch between the motor system and perception having an effect on eeriness are quite possible.

Although we tried to make experimental setting as similar as possible to the real-world situations, there still is a technical limitation such that, we only used isolated words to evaluate the perception of own voice. Spoken language consists of a series of words with various acoustic characteristics. Thus, our result may not be directly applicable to the spoken language in our daily life situations.

Despite the phenomenon that people feel creepiness when confronted with objects having a human-like appearance such as mannequins, the existence of the uncanny valley has been questioned in several experimental studies. A proportional relationship between eeriness and the human similarity was found only for digitally created faces [36]. A nonlinear curve showing a gradual valley-like shape was observed in the morphing of robot, android, and human “faces” but it was not as clear as that in the original uncanny valley theory [29,37]. Our study supported an absence of the uncanny valley [36] in own-voice perception, especially when focusing on oneself as a measure of human likeness. Future studies using a completely unfamiliar voice will examine the existence of the valley in other aspects of audition.

Our results indicate the importance of individual consideration in own voice reproduction experiments and cast doubt on the existence of the uncanny valley in terms of own voice perception. Methods for complete and genuine reproduction of own-voice may be of use to various fields. For example, listening to non-stuttering own voice may be used to treat stutter. In terms of clinical research, presentation of own voice may facilitate research on hallucination in schizophrenia. Thus, our study may act as stepping-stone for more detailed research on own-voice and perception of self across many fields.

## Supporting information

S1 Fig Results of a-posterior pitch analysis. Blue line indicates recorded voice and red line indicates adjusted voice. Results from the first six participants from experiment 1 are shown.
(EPS)

S1 Table Individual results of pairwise comparisons. The values represent numbers that the filter in the row was judged more like own voice compared to the filter in the column.
(XLSX)

S2 Table Parameters adjusted by each participant. Parameters adjusted by each subject to generate the adjusted voice.
(XLSX)

S3 Table Results of SWIPE analyses. Voice transformations with DAVID were evaluated by the SWIPE algorithm.
(XLSX)
